# De Novo Design and Synthesis of Polypeptide Immunomodulators for Resetting Macrophage Polarization

**DOI:** 10.34133/bdr.0006

**Published:** 2023-02-07

**Authors:** Na Kong, Hongru Ma, Zhongji Pu, Fengju Wan, Dongfang Li, Lei Huang, Jiazhang Lian, Xingxu Huang, Shengjie Ling, Haoran Yu, Yuan Yao

**Affiliations:** ^1^ School of Physical Science and Technology, ShanghaiTech University, 393 Middle Huaxia Road, Shanghai 201210, China.; ^2^ ZJU-Hangzhou Global Scientific and Technological Innovation Center, Zhejiang University, Hangzhou 311215, China.; ^3^ College of Chemical and Biological Engineering, Zhejiang University, Hangzhou, Zhejiang 310027, China.; ^4^ Zhejiang Lab, Hangzhou, Zhejiang 311121, China.; ^5^ School of Life Science and Technology, ShanghaiTech University, Shanghai 201210, China.; ^6^ Shanghai Clinical Research and Trial Center, Shanghai 201210, China.

## Abstract

Modulating the extracellular matrix microenvironment is critical for achieving the desired macrophage phenotype in immune investigations or tumor therapy. Combining de novo protein design and biosynthesis techniques, herein, we designed a biomimetic polypeptide self-assembled nano-immunomodulator to trigger the activation of a specific macrophage phenotype. It was intended to be made up of (​GGS​GGP​GGG​PAS​AAA​NSA​SRA​TSN​SP)*_n_*, the RGD motif from collagen, and the IKVAV motif from laminin. The combination of these domains allows the biomimetic polypeptide to assemble into extracellular matrix-like nanofibrils, creating an extracellular matrix-like milieu for macrophages. Furthermore, changing the concentration further provides a facile route to fine-tune macrophage polarization, which enhances antitumor immune responses by precisely resetting tumor-associated macrophage immune responses into an M1-like phenotype, which is generally considered to be tumor-killing macrophages, primarily antitumor, and immune-promoting. Unlike metal or synthetic polymer-based nanoparticles, this polypeptide-based nanomaterial exhibits excellent biocompatibility, high efficacy, and precise tunability in immunomodulatory effectiveness. These encouraging findings motivate us to continue our research into cancer immunotherapy applications in the future.

## Introduction

Macrophages, which are found throughout the body, are one of the most prevalent tumor-infiltrating leukocytes and are classified as either proinflammatory M1 phenotypes or anti-inflammatory M2 phenotypes [1–3]. Macrophages are a 2-edged sword in cancer therapy [4]. M1-like macrophages inhibit tumor growth and facilitate cancer therapy via secreting chemokines, such as interleukin 6 (IL6) and tumor necrosis factor α (TNFα), whereas M2-like macrophages promote tumor growth [5]. The biomaterial-mediated immune response is dependent on intricate interactions between the biomaterial and the host, including the inhibition of innate immunity and activation of immunosuppression. To enhance tumor therapy, a number of inorganic nanomaterial-based nano-immunomodulators have been developed to activate the desired macrophage phenotype [6]. For example, a variety of metallic nanoparticles (e.g., Au, Ag, Co, ZnO, and TiO_2_) [6–9] and inorganic nanomaterials (e.g., SiO_2_ nanoparticle, ferumoxytol nanoparticle, and graphene oxide nanosheet) [10–12] have been reported that can enhance macrophage polarization of RAW264.7 and J774.A1 by facilitating the phenotype transition from M0 to M1. Other inorganic nanoparticles, such as Ti with Ca and Sr surface coating, could also facilitate the transition from the M0 to the M2 polarized phenotype [13,14]. In these circumstances, the disadvantages of inorganic nanoparticles in biomedical engineering, such as biotoxicity, cell metabolism, low biocompatibility, difficulties in metabolism, and a narrow dosage window, cannot be disregarded and limit their future applicability [15,16]. For instance, Ag nanoparticles exhibit size-dependent cytotoxicity that modifies cellular antioxidant levels and arrests the cell cycle, reducing the viability and differentiation of osteoblasts and osteoclasts [15]. TiO_2_ nanoparticles induce rat hippocampal induces hippocampal neuron apoptosis by oxidative stress and inflammation [16].

Accordingly, it remains a marked challenge to develop a nano-immunomodulator with outstanding biocompatibility and precise immunomodulation capability. In this context, the remarkable cytocompatibility, controllable biodegradability, and metabolizability of proteins and polypeptide-based nanomaterials make them promising [17–20]. Biomimetic nanofibril for tumor microenvironment engineering provides a superior model for investigations of tumor mechanobiology and pathology, as well as new cancer immunotherapy approaches [21]. Abundant evidence showed that protein-based nanofibrils such as fibrin [22], collagen [23], and Matrigel [24] have shown great promise as matrices for fibrotic tissue or disease model investigation. Although these naturally derived materials match the biochemistry and fibrillar structure of the native ECM (extracellular matrix), few of them have immunomodulatory activity, lack batch-to-batch stability, and may contain xenogeneic compounds [25]. These limit the applications of immunomodulation for cancer therapy. The synthetic polymer-based nanofibrils such as polyacrylamide or poly(ethylene glycol) present significant limitations in recapitulating the fibrillar architecture of the natural fibrotic ECM. Therefore, to overcome these limitations, it is inevitable to engineer a biomimetic nanofibril for local niche immunomodulation.

In particular, the emergence of de novo protein design and synthesis technologies provides the possibility to design the structure, properties [26–30], and functions of nanomaterials at the molecular level [31–33]. Here, we design biomimetic polypeptides (BMPPs) containing sequences fused with motifs of spidroin and Arginyl-glycyl-aspartic acid (RGD) and l-isoleucine-Lysine-Valine-Alanine-Valine (IKVAV) in the “ECM.” This molecular property enables programmable self-assembly of the polypeptide from bottom-up approaches, endowing size-controllable nanofibrils. These nanofibrils, which function as nano-immunomodulators and take advantage of the biomimetic qualities of polypeptide-based nanofibrils, may accurately reset macrophages to M1 for an immunological response. In addition to having good biocompatibility, these nanofibrils as immunomodulators precisely enhance the protein expression levels of IL6 and TNFα in accordance with the BMPP dosage.

## Materials and Methods

### Design of BMPPs and structure simulation

The polypeptide design involved both self-assembly and bifunctional parts. The core sequence (ASAAANSASRATSNS)_8_ was selected from our former de novo assembly results of outer egg sac silk from *Nephila pilipes* [34]. Amino acids used are alanine (A), glycine (G), asparagine (N), proline (P), arginine (R), serine (S), and L-threonine (T). Cell-binding domains of both RGD and IKVAV were tailored to the protein terminus. Proline insertion was added for topology and stability controlling in the protein folding process. Monomer design and structure were evaluated on MSA (multiple sequence alignment).

### BMPP biosynthesis and purification

The designed polypeptide sequence was cloned into a pRSFDuet1 plasmid fused with sumo tags on its N terminus and transformed into *Escherichia coli* BL21 (DE3). The strain was cultured with Luria broth (LB; 20 ml) medium containing kanamycin (10 μl, 100 mg/ml) for 6 h at 37 °C under shaking (220 rpm) until OD_600_ (optical density at 600 nm) reached 0.6. Then, we applied isopropylthiogalactoside to induce the designed polypeptide expression, followed by precipitate collection and resuspension with lysozyme in a buffer containing 20 mM tris-HCl and 150 mM NaCl (pH = 8). After that, the resuspension solution was carried out in a high-pressure cell disrupter at 400 bar (Union, LH-03) to harvest protein. The lysate was centrifuged at 12,000g for 20 min at 4 °C, and the precipitate was dissolved in 50-ml guanidine hydrochloride (6 M).

The purification started with a 0.22-μm membrane filter. The filtrate was loaded on a nickel-charged nitrilotriacetic acid (Ni-NTA) column (HUACHUN, 5-ml high performance) eluting with 30-ml imidazole (0.5 M) in buffer containing 6 M guanidine hydrochloride, 20 mM tris-HCl, 0.5 M NaCl, and 1 mM DL-dithiothreitol. Then, the eluted protein was diluted by 3 and 1 M guanidine hydrochloride, respectively, using centrifugal filter units (Vivaspin 20, GE Healthcare; cutoff at 5,000 g). Then, the protein was substituted into the Ulp1 enzyme and buffer (25 mM tris-HCl, pH = 7, 150 mM NaCl, and 1 mM DL-dithiothreitol) at 4 °C before loading into the Ni-NTA column. The purified aqueous protein solution was dialyzed to a buffer containing 20 mM tris-HCl and 0.5 M NaCl for 24 h.

To assess the resulting protein, sodium dodecyl sulfate-polyacrylamide gel electrophoresis (SDS-PAGE; 4% to 20%; GenScript) for Coomassie Brilliant Blue staining was used to identify the BMPPs, and matrix-assisted laser desorption/ionization mass spectrometry (MALDI-MS; sinapinic acid as a matrix) was performed on a Bruker autoflex speed.

### Protein structure prediction

tFold (https://drug.ai.tencent.com/cn) modeling was used to predict the monomer structure based on the official protocols. The predicted structures were further subjected to molecular dynamics (MD) simulations. Clustering was performed using DBSCAN [35] via the cpptraj module of AmberTools, and the last 500 ns of simulation trajectory was used for clustering [36]. Protein–protein docking technique was employed to predict the assembled protein complex. The cluster model was subjected to HDOCK docking protocol [37]. Top 10 ranked docking poses were then retrieved and analyzed for structure analysis.

### MD simulation

The predicted monomers and higher-order complexes were further rigorously validated by performing MD simulations [38]. The ff14SB force field was used to describe all protein parameters: Protein was solvated using the Transferable interatomic potential with three points (TIP3P) water model with a minimum of 12 Å of padding to form a cubic periodic box and then electrically neutralized with an ionic concentration of 0.15 M NaCl. Production simulations of 600 and 100 ns were performed for monomers and higher-order complexes, respectively. AmberTools was applied for root mean square deviation (RMSD) of the backbone atoms to the initial predicted structure and the secondary structure. We employed the MM/GBSA (molecular mechanics/generalized born surface area) approach to calculate the binding free energy [39], using the implicit solvent model proposed by Hawkins et al. [40]. (corresponding to the MM/GBSA parameter igb=1 ).

### Atomic force microscopy

Atomic force microscopy (AFM) analysis was performed using the Bruker Dimension FastScan Atomic Force Microscope. Samples were characterized under the mode of ScanAsyst in air, using a ScanAsyst-Air probe. Samples were prepared by spin-coating 20-μl aliquot of the fibril solution onto clean silicon wafers. The wafers were subsequently rinsed using deionized water to remove the salt ions and dry overnight at room temperature before imaging.

### BMPP assembly and kinetics studies

The lyophilized BMPPs were dissolved in 200 mM potassium phosphate (pH = 8) and filtered with 0.22-μm membranes. The nanofibrils were subsequently prepared by stirring (500 rpm) the BMPP solution at room temperature. Kinetic experiments were conducted using turbidity measurement. Absorption at 340 nm was recorded every 10 min during the aggregation course. The assembly process:

1. Nucleation

$$A\overset{k_{1}}{\to }B$$ (1)

2. Autocatalytic surface growth

$$A+B\overset{k_{2}}{\to }2B$$ (2)

$$B+B\overset{k_{3}}{\to }2B$$ (3)

where *A* is the BMPP monomer, *B* is the nanofibril nucleus, and 2*B* is the elongated nanofibril. *k*_1_ is the rate constant of nucleation, and *k*_2_ and *k*_3_ are the rate constants of nanofibril growth by monomer addition or assembly between the nucleus. Rate constants were obtained by derivative of kinetic curves.

### Circular dichroism measurement

Circular dichroism (CD) spectra were obtained using a spectropolarimeter (Applied Photophsics, UK). The CD spectra were performed every 10 min during the aggregation course. All CD spectra were recorded using a diluted 20 μM aqueous protein solution in a 1-mm quartz cuvette.

### Cell proliferation assay

For the cell proliferation assay, we performed the EdU cell proliferation staining using an 5-ethynyl-2′- deoxyuridine (EdU) kit (BeyoClick EdU Cell Proliferation Kit with Alexa Fluor 488, Beyotime, China). Briefly, RAW264.7 cells (5 × 10^4^ cells per well) were inoculated in 96-well plates and cultured in Dulbecco’s modified Eagle’s medium (DMEM) containing BMPP for 24 h. Subsequently, cells were incubated with EdU for 3 h, fixed with 4% paraformaldehyde for 15 min, and permeabilized with 0.3% Triton X-100 for 15 min. Cells were incubated with click reaction mixture for 30 min at room temperature in the dark, followed by incubation with Hoechst 33342 for 10 min. Then, photographing and counting are done with the aid of a high-content screening system (Thermo Fisher Scientific).

### Cell differentiation assay

To verify the effect of nano-immunomodulators prepared with BMPP on macrophages, we followed the method of collagen plating and coated 24-well plates with different concentrations of BMPP. Specifically, 300 μl of different concentrations of BMPP was added to the well plates, left for 18 h at room temperature for self-assembly, washed twice with phosphate-buffered saline (PBS), and incubated with DMEM complete medium for 2 h in a 37 °C incubator. RAW264.7 cells (2 × 10^5^ cells per well) were seeded in the BMPP-coated 24-well plates and cultured in DMEM with BMPP for 48 h. RAW264.7 cells with 1000 ng/ml of lipopolysaccharide (LPS) for 24 h were used for the positive control [41].

### Immunofluorescence imaging

Cells were seeded in 96-well plates with manual BMPP coating, culturing for 48 h. After discarding the medium, cells were washed 3 times with prewarmed PBS and fixed in 4% paraformaldehyde for 15 min at room temperature. After washing 3 times with PBS, 0.3% Triton X-100 (Sangon Biotech, Shanghai, China) containing 5% goat serum (Sigma) was added and incubated for 30 min. Primary antibodies, α/β-tubulin (Cell Signaling Technology; 1:100), were diluted 1:200 with 0.3% Triton-X in 1 × PBS and incubated overnight at 4 °C. Subsequently, the cells were stained with Alexa Fluor 488–phalloidin and Alexa Fluor 647 (Cell Signaling Technology; 1:1,000) conjugated secondary antibodies containing 1% bovine serum albumin with 0.3% Triton X-100 at room temperature for 1 h. Finally, nuclei were stained with 4′,6-diamidino-2-phenylindole (DAPI). Images and subsequent analysis were done with the high-content screening system (Thermo Fisher Scientific).

### Real-time polymerase chain reaction

Cells were washed with PBS, and RNA was isolated using the RNA isolater Total RNA Extraction Reagent (Vazyme), following standard protocol. To prepare cDNA, 500 ng of RNA was prepared with a genomic DNA wiper for 5 min, and cDNA was synthesized with the HiScript II Q RT SuperMix for quantitative polymerase chain reaction (qPCR) (Vazyme). Real-time PCR reactions contained cDNA (1:3 dilution), forward and reverse primers, and the Taq Pro Universal SYBR qPCR Master Mix. Each sample was analyzed in triplicate (ABI StepOnePlus, Thermo Fisher Scientific), and relative expression was determined by 2^−ΔΔCt^, in which ΔΔCt = ΔCt (BMPP) − ΔCt (negative control), where ΔCt represents the PCR Ct of a testing molecule normalized by the Ct of actin.

### Enzyme-linked immunosorbent assay

After culturing RAW264.7 cells for 48 h, the medium was collected. Centrifuge at 800 rpm for 5 min was done to remove residual cells and to collect supernatant for enzyme linked immunosorbent assay (ELISA). ELISA assays are performed according to the kit instructions. ELISA kits include the mouse IL6-, mouse IL10-, and mouse TNFα-precoated ELISA kit.

### Statistics

Data were analyzed with one-way analysis of variance (ANOVA) on GraphPad Prism software. Significant *P* values were those less than 0.05.

## Results

### Design and synthesis of BMPPs

**Fig. 1.**
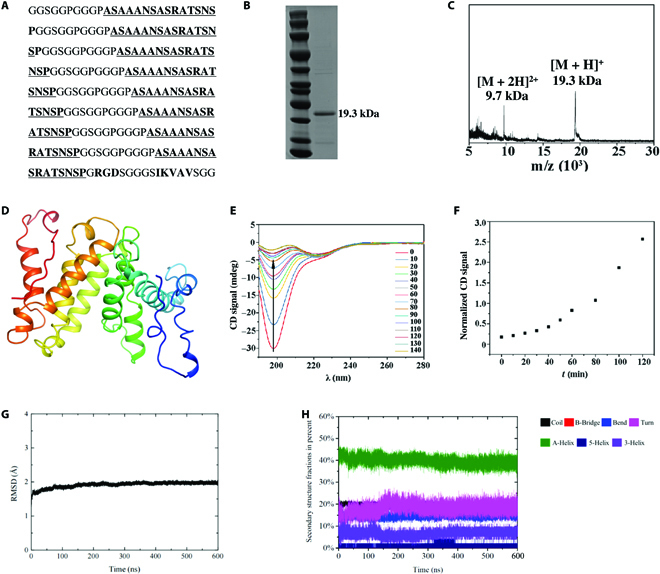
De novo design, nanofibril structure, and computational modeling of BMPP. (A) The amino acid sequence of BMPP. (B and C) BMPP characterization: (B) SDS-PAGE gel stained with Coomassie blue (unit: kDa) and (C) MALDI-MS spectrum (unit: kDa). (D) Predicted protein structure of BMPPs by tFold. (E) Circular dichroism (CD) signal of BMPPs at different assembly time points(min). (F) Intensity ratio of a-helix and random coil during BMPPs assembly. (G) Backbone RMSDs for predicted protein structure during the MD simulations. (H) Protein secondary structure content for BMPPs during the MD simulations.

We designed a fusion protein using 3 motifs to construct a tandem building block of polypeptides: (GGSGGPGGGPASAAANSASRATSNSP)*_n_*, which is derived from the de novo sequence of *N. pilipes* silk [34]; the RGD motif from collagen; and the IKVAV motif from laminin (Fig. 1A). As the backbone, the spidroin-mimicking motif of ASAAANSASRATSNS contributes to high tensile strength, whereas GGX/GPGX produces amorphous domains that contribute to extensibility fibers [42–46]. The amino acid sequence and the number (*n*) of repeated blocks of these spidroin-mimicking motifs are adjustable. For example, tandem arrayed GGX in MaSp1 or GPGX in MaSp2 and ASAAANSASRATSNS from *N. pilipes* silk are tunable for their insertion location and repeat unit within the design sequence to determine the formation capability of the nanofibril. Meanwhile, proline was inserted alternately between these motifs to influence the topology and stability of protein folding. Cell binding sites of both RGD and IKVAV from the mammalian ECM are optimized for integrin recognition to aid the putative immunomodulatory capabilities of the BMPPs. Among them, integrin β engagement can induce the activation of nuclear factor κB (NF-κB), which elevates inflammatory cytokines and results in proinflammatory polarization (M1) of macrophages [47,48]. Laminin-derived IKVAV peptides can promote beneficial cell interactions and attenuate cell contacting responses [49]. The C terminus of these functional motifs was specifically designed for immunomodulation and cell recognition. It is highlighted that even after subsequent fusion with the functional peptide motifs, which results in the generation of size-controllable nanofibrils, the designed motif’s capacity to assemble into nanofibrils can be preserved.

**Table 1.** Primer list.

Name	Sequence
m-actin-qRT-F	TGAGCGCAAGTACTCTGTGTGGAT
m-actin-qRT-R	ACTCATCGTACTCCTGCTTGCTGA
M-Arg1-F	GGAATCTGCATGGGCAACCTGTGT
M-Arg1-R	AGGGTCTACGTCTCGCAAGCCA
M-NOS2-F	CAGCTGGGCTGTACAAACCTT
M-NOS2-R	CATTGGAAGTGAAGCGTTTCG
IL-6-F	CTTGGGACTGATGCTGGTGAC
IL-6-R	TTCTCATTTCCACGATTTCCCA
IL-10-F	GCTCTTACTGACTGGCATGAG
IL-10-R	CGCAGCTCTAGGAGCATGTG
TNF-α-F	GGCGGTGCCTATGTCTCAG
TNF-α-R	GGCTACAGGCTTGTCACTCG

Experimentally, BMPPs were biosynthesized using genetically engineered *E. coli* BL21 (DE3). The detailed experimental protocol can be found in the BMPP biosynthesis and purification section. The excellent purity of the BMPPs was validated using SDS-PAGE (Fig. 1B). In addition, MALDI-MS revealed that BMPPs have 2 heterogeneous components, that is, [M + 2H]^2+^ and [M + H]^+^ with a molecular weight of 9.7 and 19.3 kDa, respectively (Fig. 1C).

The molecular structure of the BMPP monomer was then predicted using tFold (Fig. 1D) [50–52]. As expected, the BMPPs were found to be in an α-helix-rich conformation, which is formed by the repeat unit of GGSGGPGGGPASAAANSASRATSNSP. In addition, CD spectrometric measurements (Fig. 1E and F) revealed that the α-helix in BMPPs is transferred from the random coil, resulting in the negative random coil band at 198 nm progressively shifting to the α-helix band at 222 nm. The accuracy of the predicted protein structure was further improved by performing MD simulations. To obtain ensembles of representative MD conformations, the last 500-ns simulation trajectory of the structure predicted by tFold was used for clustering analysis performed using DBSCAN via the cpptraj module of AmberTools (Fig. 1G and H). More than 99% of MD conformations were grouped into one cluster, and the most representative conformation was used to study the self-assembling process.

### Self-assembly of BMPPs

**Fig. 2.**
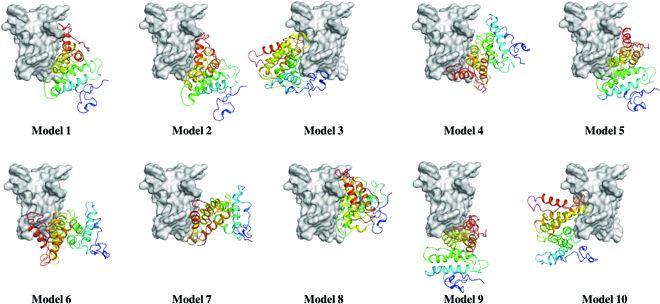
Overall representation of the protein–protein complexes through HDOCK. Top 10 models of BMPP predicted by HDOCK.

**Fig. 3.**
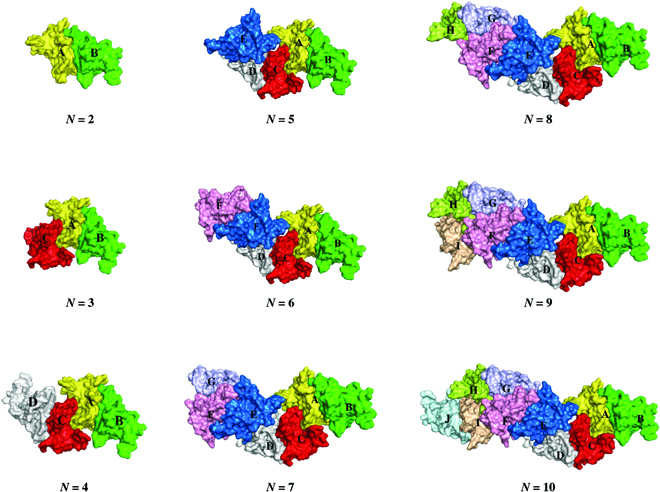
Docking models of 2nd to 10th BMPP self-assembly complexes (*N* from 2 to 10).

Protein–protein docking simulation disclosed that specific interfacial binding played a vital role in forming BMPP nanofibrils. The top 10 docking models created by HDOCK are depicted in Fig. 2, with models 1, 2, 4, 5, 6, 7, 8, and 9 showing comparable binding positions, and models 3 and 10 binding to different binding interfaces. Then, using model 1, with the lowest docking energy score, as a template, complex BMPP structures with 2 to 10 monomers were created (Fig. 3). The self-assembly models were further subjected to MD simulations to investigate the dynamics and stability, and binding analysis was performed using the MM/GBSA approach. According to the observed results (Movies S1 to S3), 3 complex models demonstrated good stability in terms of RMSD. The averaged interface binding free energy was −291.88 ± 8.37, −253.17 ± 7.75, −278.05 ± 8.55, −253.42 ± 8.11, and −251.49 ± 8.04 kcal/mol for second-, third-, fourth-, fifth-, and sixth-order assembly, respectively, indicating that the binding affinity remains steady during the assembly process (Table 2). Furthermore, the binding energy between monomers A and B appeared to be lower than that of other monomers, with the exception of the last 2 monomers, E and F in the sixth complex and C and D in the fourth complex, implying that binding affinity between monomers on each side of the nanofibril is stronger than that between monomers in the middle. Obviously, this is beneficial to the elongation of BMPP nanofibrils.

**Table 2.** The averaged interface binding free energy between BMPP monomers. (Unit: kcal/mol).

Order	*E*_AB_	*E*_AC_	*E*_CD_	*E*_DE_	*E*_EF_	*E*_FG_	*E*_GH_	*E*_HI_	*E*_IJ_
2	−226.39 ± 8.77								
3	−223.28 ± 7.85	−174.53 ± 7.25							
4	−227.95 ± 8.40	−142.84 ± 6.62	−261.49 ± 8.40						
5	−217.97 ± 6.67	−145.06 ± 10.17	−244.24 ± 7.80	−153.33 ± 5.89					
6	−225.78 ± 8.12	−142.02 ± 7.29	−192.97 ± 7.71	−148.02 ± 5.74	−233.88 ± 7.71				
7	−260.39 ± 9.03	−137.73 ± 5.71	−256.12 ± 7.85	−155.06 ± 7.88	−212.02 ± 7.91	−179.50 ± 6.58			
8	−247.44 ± 7.50	−154.48 ± 6.26	−242.33 ± 8.69	−176.80 ± 6.67	−239.32 ± 8.31	−160.23 ± 4.93	−249.14 ± 9.35		
9	−211.68 ± 8.60	−139.84 ± 6.23	−259.12 ± 9.08	−145.72 ± 6.10	−240.57 ± 8.66	−192.78 ± 8.88	−262.89 ± 7.76	−152.84 ± 5.82	
10	−266.12 ± 8.10	−137.59 ± 6.58	−199.88 ± 6.88	−141.70 ± 7.67	−235.39 ± 7.32	−178.66 ± 5.94	−222.52 ± 7.30	−166.66 ± 6.05	−223.30 ± 7.66

**Fig. 4.**
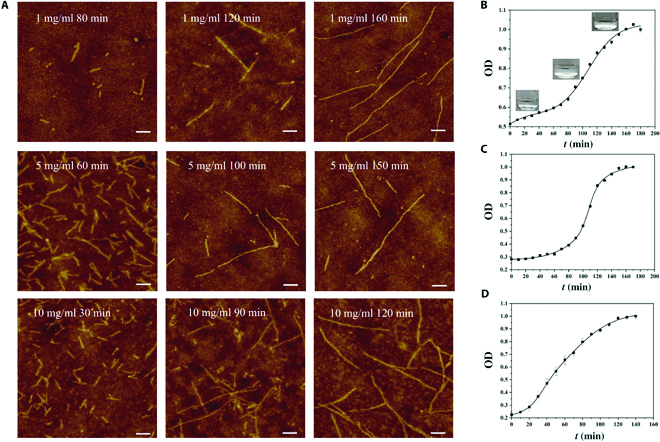
Self-assembly of polypeptide BMPPs. (A) AFM images of nanofibrils at concentrations of 1, 5, and 10 mg/ml in the process of nucleation, growth, and equilibrium, respectively. (B to D). Assembly kinetics of BMPPs at (B) 1 mg/ml, (C) 5 mg/ml, and (D) 10 mg/ml with the OD value. Scale bars, 100 nm.

**Table 3.** Aspect ratio and kinetic parameters of BMPP assembly.

*C* (mg/ml)	Nanofibrils			Rate constants		
	Diameter (nm)	Length (nm)	Aspect ratio	*k*_1_ (min^−1^)	*k*_2_ (mM・min^−1^)	*k*_3_ (mM・min^−1^)
1	10	579 ± 115	60		1.4E−3	6.5E−3	3.4E−3
5		578 ± 86	60		1.6E−3	1.6E−2	4.8E−3
10		628 ± 94	63		9.9E−3	7.4E−3	4.9E−3

The BMPPs were self-assembled at room temperature, and the detailed experimental technique may be found in the BMPP assembly and kinetics studies section [53,54]. As shown in Fig. 4A, the BMPPs were assembled into elongated single nanofibrils at concentrations ranging from 1 to 10 mg/ml. The kinetics of BMPP assembly, similar to that of other protein assemblies, follows the S-shaped double exponential increase, as shown in Fig. 4B to D. Figures S1 to S3 provide a detailed explanation of the assembly kinetics of BMPPs. The assembly kinetic assessments show that the time required for assembly gradually decreases as the concentration increases (Table 2).

In the nucleation stage, AFM reveals that the nanofibrils at the initial stage are uniform in terms of both diameter (~10 nm) and contour lengths (~70 nm). The diameter of the nanofibrils does not vary with the progressive growth of BMPPs, but their contour length increases continually, and the difference in final nanofibril length between different concentrations is not noticeable. For example, after the final growing equilibrium (Fig. 4A), the contour lengths of BMPPs are 579 ± 115, 578 ± 86, and 628 ± 94 nm (Fig. 4A and Table 3). These similarities are undeniably important for the application of BMPP nanofibrils as a nano-immunomodulator.

### BMPP nano-immunomodulators for steering the cellular gradients of macrophages

**Fig. 5.**
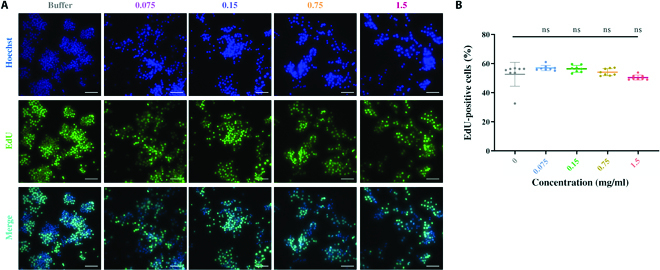
Biosafety and cytocompatibility of BMPPs. Cell proliferation was determined by (A) EdU staining, and (B) EdU incorporation was calculated as EdU^+^ cells/total cells, quantified by the high-content screening system. The experiments were repeated 6 times with reproducible results; statistical significance was assessed by one-way ANOVA; ns, *P* > 0.05 compared with the control. Error bars denote mean ± SD. Scale bars, 100 μm.

As previously stated, BMPPs can self-assemble into nanofibrils with the usual topology of ECM. We also attempted to evaluate its biofunctionality as a nano-immunomodulator. Because the biosafety and cytocompatibility of BMPPs are necessary, the cell proliferation rate of RAW264.7 with a moderate dose of BMPPs was studied. The cell proliferation rate was measured as EdU^+^ cells/total cells and was statistically quantified by the high-content screening system. As shown in Fig. 5A and B, consistent with the control group (the buffer without BMPPs), BMPPs with concentrations ranging from 0.075 to 1.5 mg/ml maintain a constant proliferation rate of around 55%, supporting the biosafety and cytocompatibility of BMPPs.

**Fig. 6.**
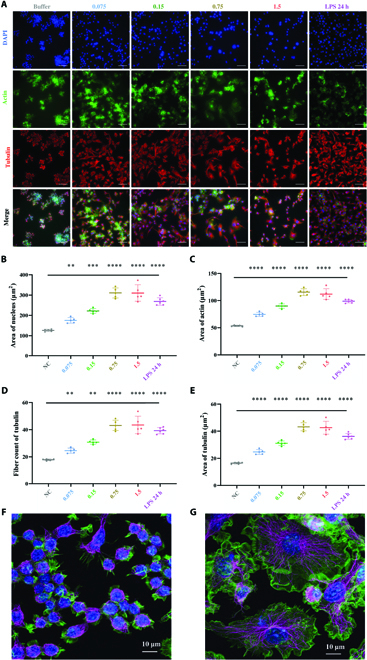
BMPPs steer the cellular gradients of the macrophage of RAW264.7. (A) Immunofluorescence staining of cultured cells with DAPI (blue), actin (green), and tubulin (red). DAPI is for staining the cell nuclei, and actin and tubulin are stained to show the cytoskeleton in cells. Scale bars, 100 μm. (B) Changes in cell nuclei after culturing in different concentrations of BMPPs (unit: mg/ml). (C to E) Changes in cell cytoskeleton after culturing in different concentrations of BMPPs (unit: mg/ml). The experiments were repeated 6 times with reproducible results, quantified by the high-content screening system; statistical significance was assessed by one-way ANOVA; ****P < 0.0001, ***P < 0.001, and **P < 0.01. Error bars denote mean ± SD. (F and G) Morphology of macrophages in the NC group (F) and 1.5 mg/ml of BMPP group (G).

To investigate how RAW264.7 adapts to the BMPP nano-immunomodulator, the corresponding cellular gradients of RAW264.7 cells were characterized by immunostaining using a high-content screening system. The findings revealed that the BMPP nano-immunomodulators direct RAW264.7 cells into a progressive shift as a morphological feature, including nuclear area and cytoskeleton distribution (Fig. 6A to E). RAW264.7 cells, for example, demonstrated a steady morphing pattern from 0.075 to 1.5 mg/ml as continuously enhanced changes in cell morphology, with the associated nucleus area gradually shifting from 175.5 to 310.3 μm^2^ (Fig. 6B). RAW264.7 morphology was continuously and gradually elongated by cytoskeleton reorganization, as illustrated in Fig. 6A, F, and G. Typically, the majority of RAW264.7 cells in the 1.5 mg/ml group had changed morphology. In comparison, cells on the surface control group exhibited a spheroidal morphology, which is characterized by a smaller spreading shape with intersecting actin and tubulin filaments (Fig. 6A and C to G).

### BMPP nano-immunomodulators precisely reset macrophage immune response

**Fig. 7.**
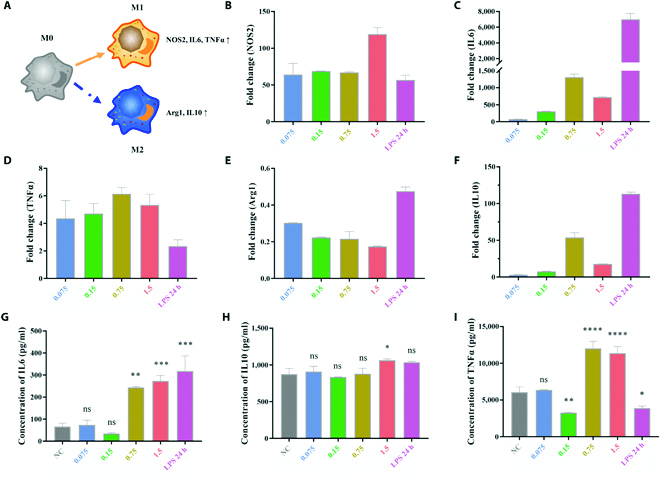
BMPPs induce macrophage polarization toward an M1-like phenotype. (A) The cartoon schematic of macrophage polarization. (B to F) Cytokine mRNA was quantified by real-time PCR. Expression of IL6, IL10, and TNFα protein levels was detected by ELISA (G to I). The experiments were repeated 3 times with reproducible results; statistical significance was assessed by one-way ANOVA; ns, *P* > 0.05; ****P < 0.0001, ***P < 0.001, **P < 0.01, and *P < 0.05. Error bars denote mean ± SD.

Cytoskeleton reorganization and nuclear deformation may be related to macrophage polarization [55]. In order to evaluate the macrophage phenotype using up-regulated gene expression and related protein levels, qPCR and ELISA were used. We assessed RAW264.7 inflammatory cytokine secretion in response to higher BMPP concentrations. The activation level of M1 macrophages was measured using LPS. M1 macrophage activation increases the levels of nitric oxide synthase 2 (NOS2), IL6, and TNF, whereas M2 macrophage polarization increases the production of arginase 1 (Arg1), and IL10. NOS2, IL6, and TNFα gene expressions were significantly increased, as indicated in Fig. 7B to F. For example, the expression of NOS2 on 1.5 mg/ml of BMPP nano-immunomodulators was ~120-fold, even higher than that of the LPS group (Fig. 7B). The expression of TNFα on 0.75 mg/ml of BMPP nano-immunomodulators was ~6-fold, also higher than that of the LPS group (Fig 7D). In contrast, the expression of Arg1 was down-regulated 3- to 5-fold in different concentrations (Fig. 7E). In addition, ELISA results showed that BMPP nano-immunomodulators correspondingly increased the protein expression levels of IL6 and TNFα (Fig. 7G and I), whereas expression of IL10 has no obvious change compared to the control groups (Fig. 7H). Both findings show that M1 macrophage polarization occurs at high BMPP concentrations (i.e., 1.5 and 0.75 mg/ml) and that the BMPP nano-immunomodulators may accurately reset macrophage immune response.

## Discussion

Reprogramming the tumor microenvironment by modulating macrophages holds excellent promise in tumor immunotherapy [56]. However, the nano-immunomodulators that have so far been created, such as metal nanoparticles and other inorganic nanomaterials, continue to confront hurdles in the biosafety and narrow dosage window for immunomodulating effects. In this case, this study developed a protein/polypeptide-based nano-immunomodulator by combining de novo protein design and biosynthesis techniques. Unlike metal or synthetic polymer-based nanoparticles, polypeptide-based nanomaterial BMPPs exhibit great cytocompatibility, high efficacy, and precise tunability in immunomodulatory effectiveness.

This protein is made up of 2 fundamental building blocks, i.e., ASAAANSASRATSNS and GGX/GPGX. The former, derived from *N. pilipes* silk, is used for high-tensile strength nanofibrils, whereas tandem-arranged GGX in MaSp1 or GPGX in MaSp2 is used for amorphous domains that contribute to fiber extensibility. When these 2 key structural components are combined, the resulting materials can be assembled into elongated nanofibrils. The dimension of these nanofibrils is mimicking the physical morphology of the natural ECM. These designs, which benefit the side chain motifs such as RGD and IKVAV on these biomimicking nanofibrils, can thus be utilized directly as nano-immunomodulators. Previous studies have shown that cell binding sites of both RGD and IKVAV from the ECM are optimized for integrin recognition to aid the putative immunomodulatory capabilities [47,48]. Among them, integrin β engagement can induce the activation of NF-κB and related cascading pathway, which can rise the levels of inflammatory cytokines and results in proinflammatory polarization (M1) of macrophages [47,48]. Moreover, laminin-derived IKVAV peptides can promote beneficial cell interactions and attenuate cell contacting responses [49]. These solutions work together and might enhance the efficacy of macrophage response.

Accordingly, our findings demonstrate that macrophage behavior can be modulated by regulating the abundance of the BMPP nano-immunomodulator with integrin-related motifs (i.e., RGD and IKVAV). Furthermore, the BMPP nano-immunomodulator is highly effective because of its exact tunability in promoting M1 polarization. To achieve a more beneficial effect in tumor therapy, this M1 phenotype is typically thought to be tumor-killing macrophages, primarily antitumor, and immune-promoting. Therefore, altering macrophage responses by varying amounts of subcutaneous materials with the noticeable effect is likely due to integrin binding effects as a more widespread impact of the RGD and IKVAV motif, which can bind to a wide range of integrins [57] and induce appropriate cell adhesion and aggregation. In addition, the operational dosage window for immunomodulation was significantly expanded by this type of gradient immune response offered by the BMPP nano-immunomodulator.

As a result, by recognizing and controlling the progressive changes in the immune microenvironment, it is possible to customize certain nano-immunomodulators. This study introduces a novel de novo polypeptide-based nanofibril that precisely resets macrophage polarization, which may potentially advance research in cancer immunotherapeutics.

## Data Availability

The data that support the findings of this study are available in the Supplementary Materials of this article. Supporting Information: Supporting Information is available from the Online Library or from the author.
